# Digital Biologically Plausible Implementation of Binarized Neural Networks With Differential Hafnium Oxide Resistive Memory Arrays

**DOI:** 10.3389/fnins.2019.01383

**Published:** 2020-01-09

**Authors:** Tifenn Hirtzlin, Marc Bocquet, Bogdan Penkovsky, Jacques-Olivier Klein, Etienne Nowak, Elisa Vianello, Jean-Michel Portal, Damien Querlioz

**Affiliations:** ^1^C2N, Univ Paris-Sud, Université Paris-Saclay, CNRS, Palaiseau, France; ^2^Aix Marseille Univ, Université de Toulon, CNRS, IM2NP, Marseille, France; ^3^CEA, LETI, Grenoble, France

**Keywords:** binarized neural networks, resistive memory, memristor, in-memory computing, biologically plausible digital electronics, ASICs

## Abstract

The brain performs intelligent tasks with extremely low energy consumption. This work takes its inspiration from two strategies used by the brain to achieve this energy efficiency: the absence of separation between computing and memory functions and reliance on low-precision computation. The emergence of resistive memory technologies indeed provides an opportunity to tightly co-integrate logic and memory in hardware. In parallel, the recently proposed concept of a Binarized Neural Network, where multiplications are replaced by exclusive NOR (XNOR) logic gates, offers a way to implement artificial intelligence using very low precision computation. In this work, we therefore propose a strategy for implementing low-energy Binarized Neural Networks that employs brain-inspired concepts while retaining the energy benefits of digital electronics. We design, fabricate, and test a memory array, including periphery and sensing circuits, that is optimized for this in-memory computing scheme. Our circuit employs hafnium oxide resistive memory integrated in the back end of line of a 130-nm CMOS process, in a two-transistor, two-resistor cell, which allows the exclusive NOR operations of the neural network to be performed directly within the sense amplifiers. We show, based on extensive electrical measurements, that our design allows a reduction in the number of bit errors on the synaptic weights without the use of formal error-correcting codes. We design a whole system using this memory array. We show on standard machine learning tasks (MNIST, CIFAR-10, ImageNet, and an ECG task) that the system has inherent resilience to bit errors. We evidence that its energy consumption is attractive compared to more standard approaches and that it can use memory devices in regimes where they exhibit particularly low programming energy and high endurance. We conclude the work by discussing how it associates biologically plausible ideas with more traditional digital electronics concepts.

## 1. Introduction

Through the progress of deep learning, artificial intelligence has made tremendous achievements in recent years. Its energy consumption in graphics or central processing units (GPUs and CPUs) remains, however, a considerable challenge, limiting its use at the edge and raising the question of the sustainability of large-scale artificial intelligence-based services. Brains, by contrast, manage intelligent tasks with highly reduced energy usage. One key difference between GPUs and CPUs on the one hand and brains on the other hand is how they deal with memory. In GPUs and CPUs, memory and arithmetic units are separated, both physically and conceptually. In artificial intelligence algorithms, which require large amounts of memory access, considerably more energy is spent moving data between logic and memory than in doing actual arithmetic (Pedram et al., 2017). In brains, by contrast, neurons—which implement most of the arithmetic—and synapses—which are believed to store long-term memory—are entirely colocated. A major lead for reducing the energy consumption of artificial intelligence is therefore to imitate this strategy and design non-von Neumann systems where logic and memory are merged (Indiveri and Liu, 2015; Querlioz et al., 2015; Editorial, 2018; Yu, 2018). There is new interest in this idea today with the advent of novel nanotechnology-based non-volatile memories, which are compact and fast and can be embedded at the core of the Complementary Metal Oxide Semiconductor (CMOS) process (Prezioso et al., 2015; Sa¨ıghi et al., 2015; Wang et al., 2015; Covi et al., 2016; Serb et al., 2016; Ambrogio et al., 2018; Yu, 2018). Another key difference between processors and the brain is the basic nature of computations. GPUs and CPUs typically perform all neural network computations with precise floating-point arithmetic. In brains, most of the computation is done in a low-precision analog fashion within the neurons (Klemm and Bornholdt, 2005; Faisal et al., 2008), resulting in asynchronous spikes as an output, which is therefore binary. A second idea for cutting the energy consumption of artificial intelligence is therefore to design systems that operate with much lower-precision computation.

In recent years, considerable research has been conducted to implement neural networks using analog resistive memory as synapses—the device conductance implementing the synaptic weights. To a large extent, neural network computation can be done using analog electronics: weight/neuron multiplication is performed based on Ohm's law, and addition is natively implemented with Kirchoff's current law (Prezioso et al., 2015; Serb et al., 2016; Ambrogio et al., 2018; Li et al., 2018; Wang et al., 2018). This type of implementation is, to a certain extent, very biologically plausible, as it reproduces the two strategies mentioned above. The challenge of this implementation, however, is that it requires relatively heavy analog or mixed-signal CMOS circuitry such as operational amplifiers or Analog-to-Digital Converters, resulting in significant area and energy overhead.

In parallel, a novel class of neural networks has recently been proposed—Binarized Neural Networks (or the closely related XNOR-NETs) (Courbariaux et al., 2016; Rastegari et al., 2016). In these neural networks, once trained, synapses as well as neurons assume only binary values, meaning +1 or −1. These neural networks therefore have limited memory requirements and also rely on highly simplified arithmetic. In particular, multiplications are replaced by one-bit exclusive NOR (XNOR) operations. Nevertheless, Binarized Neural Networks can achieve near state-of-the-art performance on vision tasks (Courbariaux et al., 2016; Rastegari et al., 2016; Lin et al., 2017) and are therefore extremely attractive for realizing inference hardware. The low precision of Binarized Neural Networks and in particular the binary nature of neurons—which is reminiscent of biological neurons spikes—also endows them with biological plausibility: they can indeed be seen as a simplification of spiking neural networks.

Great effort has been devoted to developing hardware implementations of Binarized Neural Networks. Using nanodevices, one natural intuition would be to adopt the strategy proposed for conventional neural networks and perform arithmetic in an analog fashion using Kirchoff's law (Yu et al., 2016; Yu, 2018). However, Binarized Neural Networks are very digital in nature and are multiplication-less. These networks can therefore provide an opportunity to benefit, at the same time, from both bioinspired ideas and the achievements of Moore's law and digital electronics. In this work, we propose a fully digital implementation of binarized neural networks incorporating CMOS and nanodevices, and implementing the biological concepts of tight memory and logic integration, and low-precision computing. As memory nanodevices, we use hafnium oxide-based resistive random access memory (OxRAM), a compact and fast non-volatile memory cell that is fully compatible with the CMOS process (Grossi et al., 2016).

However, one significant challenge to implementing a digital system with memory nanodevices is their inherent variability (Ielmini and Wong, 2018; Ly et al., 2018), which causes bit errors. Traditional memory applications employ multiple error-correcting codes (ECCs) to solve this issue. ECC decoding circuits have large areas and high energy consumption (Gregori et al., 2003) and add extra time to data access due to syndrome computation and comparison. Moreover, the arithmetic operations of error-syndrome computation are actually more complicated than those of a Binarized Neural Network. This solution is difficult to implement in a context where memory and logic are tightly integrated, especially when part of the computation is performed during sensing. This is one of the main reasons that the state of the art of RRAM for in-memory computing does not correct errors and is not compatible with technologies with errors (Chen et al., 2017, 2018). In this paper, we introduce our solution. We design, fabricate, and test a differential oxide-based resistive memory array, including all peripheral and sensing circuitry. This array, based on a two-transistor/two-resistor (2T2R) bit cell, inherently reduces bit errors without the use of ECC, and we show that it is particularly well-adapted for in-memory computing. We then design and simulate a fully binarized neural network based on this memory array. We show that the XNOR operations can be integrated directly within the sense operation of the memory array and that the resulting system can be highly energy efficient. Based on neural networks on multiple datasets (MNIST, CIFAR-10, ImageNet, and ECG data analysis), we evaluate the number of bit errors in the memory that can be tolerated by the system. Based on this information, we show that the memory nanodevices can be used in an unconventional programming regime, where they feature low programming energy (less than five picoJoules per bit) and outstanding endurance (billions of cycles).

Partial and preliminary results of this work have been presented at a conference (Bocquet et al., 2018). This paper adds additional measurements of OxRAMs with shorter programming pulses, an analysis of the impact of bit errors on more datasets (ImageNet and ECG data analysis), and a detailed comparison and benckmarking of our approach with processors, non-binarized ASIC neural networks, and analog RRAM-based neural networks.

## 2. Materials and Methods

### 2.1. Differential Memory Array for In-memory Computing

**Figure 1:**
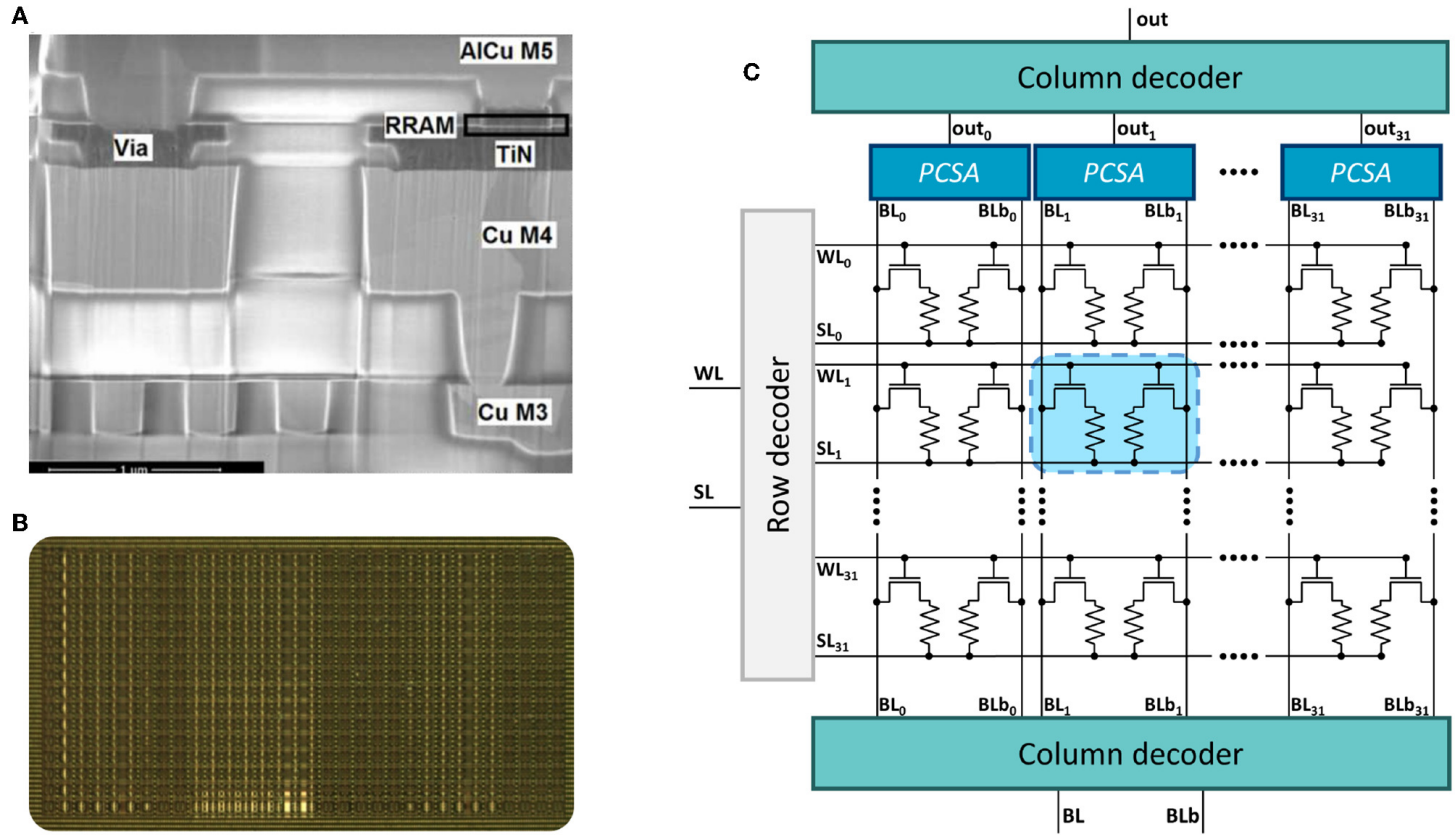
**(A)** Scanning Electron Microscopy image of the back-end-of-line of the CMOS process integrating an OxRAM device. **(B)** Photograph and **(C)** simplified schematic of the one kilobit in-memory computing-targeted memory array characterized in this work.

In this work, we fabricated a memory array for in-memory computing with its associated peripheral and sensing circuits. The memory cell relies on hafnium oxide (HfO_2_) oxide-based resistive Random Access Memory (OxRAM). The stack of the device is composed of a HfO_2_ layer and a titanium layer. Both layers have a thickness of ten nanometers, and they grow between two titanium nitride (TiN) electrodes. Our devices are embedded within the back-end-of-line of a commercial 130-nm CMOS logic process (Figure 1A), allowing tight integration of logic and non-volatile memory (Grossi et al., 2016). The devices are integrated on top of the fourth (copper) metallic layer.

We chose hafnium oxide OxRAMs because they are known to provide non-volatile memories compatible with the modern CMOS process and only involve foundry-friendly materials and process steps.

After a one-time forming process, such devices can switch between low-resistance and high-resistance states (LRS and HRS) by applying positive or negative electrical pulses, respectively. Our work could be reproduced with other types of memories. NOR flash cells, which are readily available in commercial processes, could be used, and their potential for neuromorphic inference has been proven Merrikh-Bayat et al. (2017). However, they suffer from high programming voltages (higher than ten volts) requiring charge pumps, have limited endurance, and are not scalable to the most advanced technology nodes (Dong et al., 2017). Emerging memories such as phase change memory or spin torque magnetoresitive memory could also be used adopting the strategies presented in this paper. These technologies do not require a forming process, and they can bring enhanced reliability with regards to OxRAMs but come with an increased process cost (Chen, 2016).

Conventionally, OxRAMs are organized in a “One Transistor-One Resistor” structure (1T1R), where each nanodevice is associated with one access transistor (Chen, 2016). The LRS and HRS are used to mean the zero and one logic values or the inverse. The read operation is then achieved by comparing the electrical resistance of the nanodevice to a reference value intermediate between the typical values of resistances in HRS and LRS. Unfortunately, due to device variability, OxRAMs are prone to bit errors: the HRS value can become lower than the reference resistance, and the LRS value can be higher than the reference resistance. The device variability includes both device-to-device mismatch and the fact that, within the same device, the precise value of HRS and LRS resistance changes at each programming cycle (Grossi et al., 2018).

To limit the number of bit errors, in this work, we fabricated a memory array with a “Two Transistors-Two Resistors” structure (2T2R), where each bit of information is stored in a pair of 1T1R structures. A photograph of the die is presented in Figure 1B and its simplified schematic in Figure 1C. Information is stored in a differential fashion: the pair LRS/HRS means logic value zero, while the pair HRS/LRS means logic value one. In this situation, readout is performed by comparing the resistance values of the two devices. We therefore expect bit errors to be less frequent, as a bit error only occurs if a device programmed in LRS is more resistive than its complementary device programmed in HRS. This concept of 2T2R memory arrays has already been proposed, but its benefits in terms of bit error rate have never been demonstrated until this work (Hsieh et al., 2017; Shih et al., 2017).

The programming of devices in our array is made sequentially, i.e., on a device-by-device basis. The first time that the memory array is used, all devices are “formed.” To form the device of row *i* and column *j*, the bit line *BL*_*j*_, connected to the bottom electrode of the memory device, is set to ground, and the word line *WL*_*i*_ is set to a voltage chosen to limit the current to a “compliance value” of 200μ*A*. A voltage ramp is applied to the sense line *SL*_*i*_ connected to the top electrode of the memory device, increasing from 0 to 3.3*V* at a ramp rate of 1,000 *V*/*s*. This forming operation is performed only once over the lifetime of the device. To program a device into its LRS (SET operation), the bit line *BL*_*j*_ is set to ground, while the sense line *SL*_*i*_ is set to 2*V*. The word line *WL*_*i*_ is again set to a voltage chosen to limit the current to a compliance value, ranging from 20 to 200μ*A* depending on the chosen programming condition. To program a device into its HRS (RESET operation), a voltage of opposite sign needs to be applied to the device, and the current compliance is not needed. The sense line *SL*_*i*_ is therefore set to the ground, while the word line *WL*_*i*_ is set to a value of 3.3*V*, and the bit line *BL*_*j*_ to a “RESET voltage” ranging from 1.5 to 2.5*V* depending on the chosen programming condition. For both SET and RESET operations, the programming duration can range from 0.1 to 100μ*s*. During programming operations, all bit, select, and word lines corresponding to non-selected devices are grounded, with the exception of the bit line of the complementary device of the selected device: this one is programmed to the same voltage as the one applied to the sense line to avoid any disturbing effect on the complementary device.

**Figure 2:**
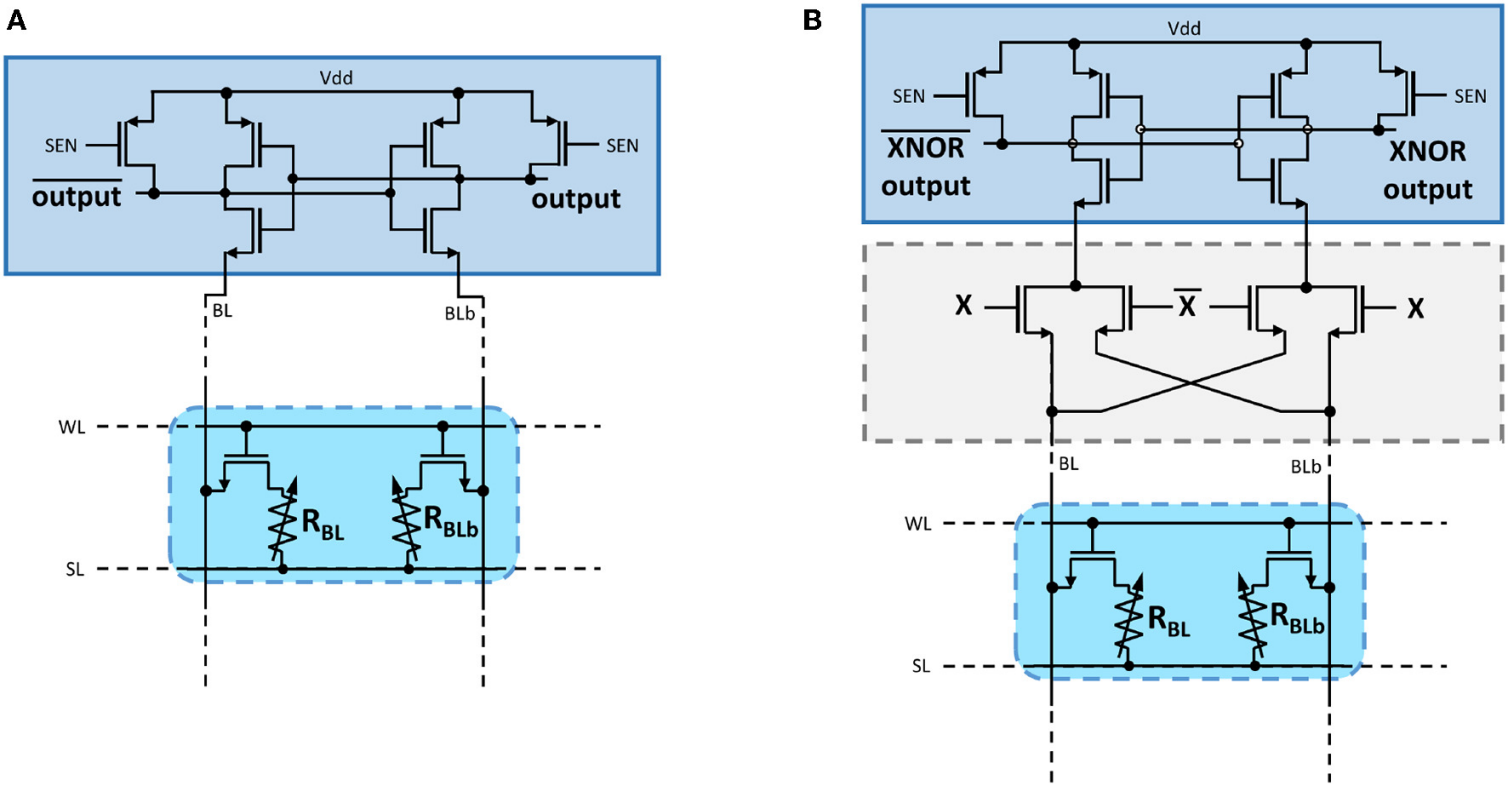
**(A)** Schematic of the precharge sense amplifier used in this work to read 2T2R memory cells. **(B)** Schematic of the precharge sense amplifier augmented with an XNOR logic operation.

In our fabricated circuit, the readout operation is performed with precharge sense amplifiers (PCSA) (Zhao et al., 2009, 2014) (Figure 2A). These circuits are highly energy-efficient due to their operation in two phases, precharge and discharge, avoiding any direct path between the supply voltage and ground. First, the sense signal (SEN) is set to ground and SL to the supply voltage, which precharges the two selected complementary nanodevices as well as the comparing latch at the same voltage. In the second phase, the sense signal is set to the supply voltage, and the voltages on the complementary devices are discharged to ground through SL. The branch with the lowest resistance discharges faster and causes its associated inverter output to discharge to ground, which latches the complementary inverter output to the supply voltage. The two output voltages therefore represent the comparison of the two complementary resistance values. In our test chip, the read time is approximately 10*ns* and results from the high capacitive load associated with our probe testing setup. Without this high capacitive load, the switching time would be determined by the time to resolve the initial metastability of the circuit. This switching time can be as fast as 100 *ps* in a scaled technology (Zhao et al., 2014).

We fabricated a differential memory array with 2,048 devices, therefore implementing a kilobit memory array. Each column of complementary nanodevices features a precharge sense amplifier, and rows and columns are accessed through integrated CMOS digital decoders. The pads of the dies are not protected from electrostatic discharge, and the dies were tested with commercial 22-pad probe cards. In all the experiments, voltages are set using a home-made printed circuit board, and pulse voltages are generated using Keysight B1530A pulse generators. In the design, the precharge sense amplifiers can optionally be deactivated and bypassed, which allows the nanodevice resistance to be measured directly through external precision source monitor units (Keysight B1517a).

### 2.2. Design of In-memory Binarized Neural Network Based on the Differential Memory Building Block

This work aims at implementing Binarized Neural Networks in hardware. In these neural networks, the synaptic weights, as well as the neuronal states, can take only two values, +1 and −1, while these parameters assume real values in conventional neural networks. The equation for neuronal value *A*_*j*_ in a conventional neural network is:

(1) $$\begin{array}{r} A_{j}=f\left(\underset{i}{\sum }W_{ji}X_{i}+b_{j}\right), \end{array}$$

where *X*_*i*_ are the neuron inputs, *W*_*ji*_ the synaptic weights values, *b*_*j*_ a bias term, and *f* the neural activation function, which introduces non-linearity into the network. Typical examples of activation functions are the sigmoid function, the softmax function, and the hyperbolic tangent function. In Binarized Neural Networks, the activation function is much simpler, as it is substituted by the sign function, as shown in Equation (2):

(2) $$\begin{array}{r} A_{j}=\text{sign}\left(\underset{i}{\mathrm{POPCOUNT}}\left(XNOR\left(W_{ji},X_{i}\right)\right)-T_{j}\right). \end{array}$$

In this equation, *T*_*j*_ is the so-called threshold of the neuron, and it is learned during training. POPCOUNT is the function that counts the number of ones in a series of bits, and sign is the sign function.

The training process of binarized neural networks differs from that of conventional neural networks. During training, the weights assume real weights in addition to the binary weights, which are equal to the sign of the real weights. Training employs the classical error backpropagation equations with several adaptations. The binarized weights are used in the equations of both the forward and the backward passes, but the real weights change as a result of the learning rule (Courbariaux et al., 2016). Additionally, as the activation function of binarized neural networks is the sign function and is not differentiable, we consider the sign function as the first approximation of the hardtanh function,

(3) $$\begin{array}{r} \text{Hardtanh}\left(\text{x}\right)=\text{Clip}\left(\text{x},-1,1\right), \end{array}$$

and we use the derivative of this function as a replacement for the derivative of the sign function in the backward pass. This replacement is a key element for training BNN successfully. The clip interval in Equation (3) is not learned and is chosen to be between −1 and 1 for all neurons. Using a larger interval would indeed increase the vanishing gradient effect, while using a smaller interval would lead to derivatives higher than one, which can cause exploding gradient effects.

Finally, the Adam optimizer is used to stabilize learning (Kingma and Ba, 2014). A technique known as batch-normalization is employed at each layer of the neural network (Ioffe and Szegedy, 2015). Batch-normalization shifts and scales the neuronal activations over a batch during the training process. This method is used optionally in conventional neural networks to accelerate and stabilize learning. Using this technique becomes essential when training binarized neural networks to reach high accuracies, as it ensures that neuronal activations utilize both +1 and −1 values. At inference time, batch-normalization is no longer necessary, and the threshold learned by this technique can be used directly as the neuronal threshold in Equation (2).

With this learning technique, binarized neural networks function surprisingly well. They can achieve near state-of-the-art performance on image recognition tasks such as CIFAR-10 and ImageNet (Lin et al., 2017). After learning, the real weights serve no more purpose and can be discarded. This makes binarized neural networks exceptional candidates for hardware implementation of neural network inference. Not only are their memory requirements minimal (one bit per neuron and synapse), but their arithmetic is also vastly simplified. Multiplication operations of Equation (1) are expensive in terms of area and energy consumption, and they are replaced by one-bit exclusive NOR (XNOR) operations in Equation (2). Additionally, the real sums in Equation (1) are replaced by POPCOUNT operations, which are equivalent to integer sums with a low bit width.

It is possible to implement ASIC Binarized Neural Networks with solely CMOS (Ando et al., 2017; Bankman et al., 2018). However, a more optimal implementation would rely on emerging non-volatile memories and associate logic and memory as closely as possible. This approach can provide non-volatile neural networks and eliminate the von Neumann bottleneck entirely: the nanodevices can implement the synaptic weights, while the arithmetic can be done in CMOS. Most of the literature proposing the use of emerging memories as synapses relies on an ingenious technique to perform the multiplications and additions of Equation (1) that relies on analog electronics: the multiplications are done based on Ohm's law and the addition based on Kirchoff's current law (Yu et al., 2016; Ambrogio et al., 2018). This analog approach can be transposed directly to binarized neural networks (Tang et al., 2017; Sun et al., 2018a,b; Yu, 2018). However, binarized neural networks are inherently digital objects that rely, as previously remarked, on simple logic operation: XNOR operations and low bit-width sums. Therefore, here, we investigate their implementation with purely digital circuitry. This concept also recently appeared in Natsui et al. (2018) and Giacomin et al. (2019) and in our preliminary version of this work (Bocquet et al., 2018). Our work is the first one to present measurements on a physical memory array that include the effect of bit errors.

A first realization is that the XNOR operations can be realized directly within the sense amplifiers. For this, we follow the pioneering work of Zhao et al. (2014), which shows that a precharge sense amplifier can be enriched with any logic operation. In our case, we can add four additional transistors in the discharge branches of a precharge sense amplifier (Figure 2B). These transistors can prevent the discharge and allow the implementation of the XNOR operation between input voltage *X* and the value stored in the complementary OxRAM devices in a single operation.

**Figure 3:**
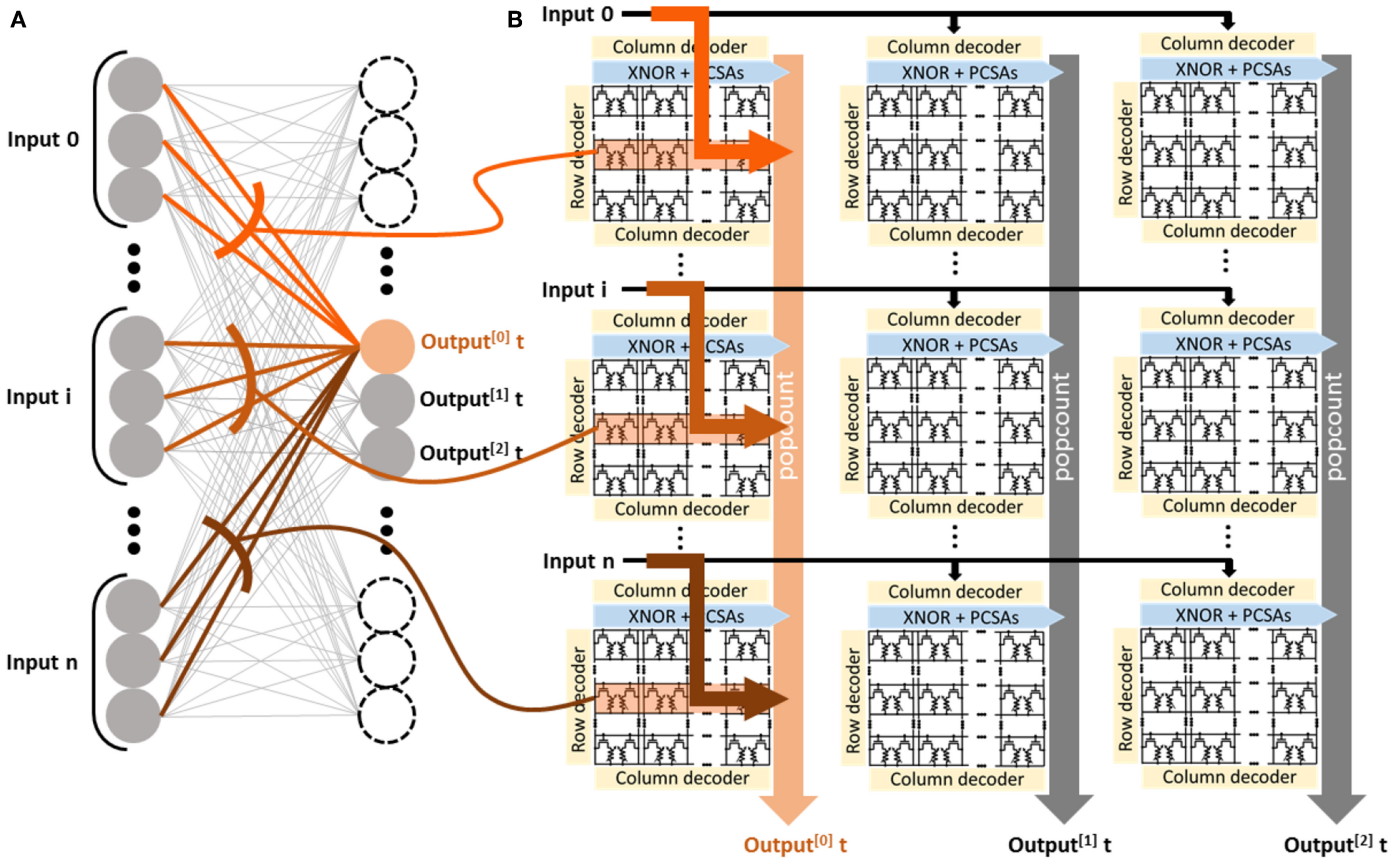
**(A)** Schematization of the implemented Binarized Neural Network highlighting connections to one specific neuron. **(B)** Schematization of the full architecture to implement the Binarized Neural Network in the “parallel to sequential” configuration. The system assembles a memory block surrounded by logic circuits and moves minimal data between the blocks. The architecture is presented with three rows and three columns (i.e., *N* = *M* = 3) of kilobit memory blocks (i.e., *n* = 32).

Based on the basic memory array with PCSAs enriched with XNOR, we designed the whole system implementing a Binarized Neural Network. The overall architecture is presented in Figure 3. It is inspired by the purely CMOS architecture proposed in Ando et al. (2017), adapted to the constraints of OxRAM. The design consists of the repetition of basic cells organized in a matrix of *N* by *M* cells. These basic cells incorporate a *n* × *n* OxRAM memory block with XNOR-enriched PCSAs and POPCOUNT logic. The whole system, which aims at computing the activation of neurons (Equation 2), features a degree of reconfigurability to adapt to different neural network topologies: it can be used either in a “parallel to sequential” or in a “sequential to parallel” configuration.

The parallel to sequential configuration (presented in Figure 3) can deal with layers with up to *n* × *N* input neurons and up to *n* × *M* output neurons. In this situation, at each clock cycle, the system computes the activations of *M* output neurons in parallel. At each clock cycle, each basic cell reads an entire row of its OxRAM memory array while performing the XNOR operation with input neuron values. The results are used to compute the POPCOUNT operation over a subset of the indices *i* in Equation (2), using fully digital five-bit counters embedded within the cell. Additional logic, called “popcount tree” and only activated in this configuration, computes the full POPCOUNT value operation over a column by successively adding the five-bit-wide partial POPCOUNT values. The activation value of the neuron is obtained by subtracting the complete POPCOUNT value at the bottom of the column from a threshold value stored in a separate memory array; the signed bit of the result gives the activation value. At the next clock cycle, the next rows in the OxRAM memory arrays are selected, and the activations of the next *M* neurons are computed.

The sequential to parallel configuration (not presented), by contrast, can be chosen to deal with a neural network layer with up to *n*^2^ inputs neurons and up to *NM* output neurons. In this configuration, each basic cell of the system computes the activation of one neuron *A*_*j*_. The input neurons *X*_*i*_ are presented sequentially by subsets of *n* inputs. At each clock cycle, the digital circuitry therefore computes only a part of Equation (2). The partial POPCOUNT is looped to the same cell to compute the whole POPCOUNT sequentially. After the presentations of all inputs, the threshold is subtracted, the binary activation is extracted, and Equation (2) has been entirely computed.

This whole system was designed using synthesizable SystemVerilog. The memory blocks are described in behavioral SystemVerilog. We synthesized the system using the 130-nm design kit used for fabrication, as well as using the design kit of an advanced commercial 28-nm process for scaling projection.

All simulations reported in the results sections were performed using Cadence Incisive simulators. The estimates for system-level energy consumption were obtained using the Cadence Encounter tool. We used Value Change Dump (VCD) files extracted from simulations of practical tasks so that the obtained energy values would reflect the operation of the system realistically.

## 3. Results

### 3.1. Differential Memory Allows Memory Operation at Reduced Bit Error Rate

**Figure 4:**
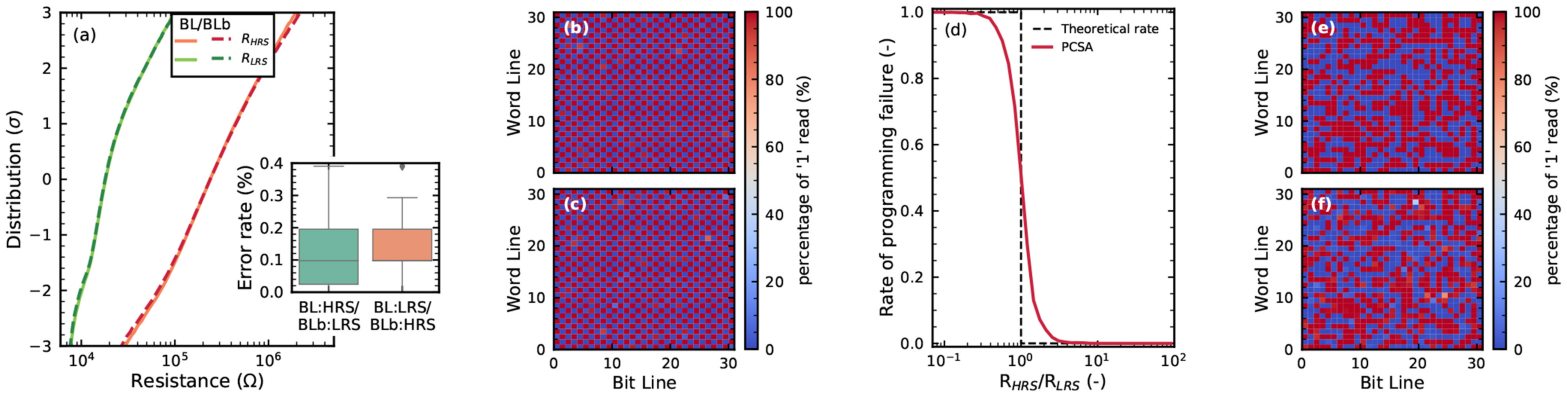
**(a)** Distributions of the LRS and HRS of the OxRAM devices in an array programmed with a checkerboard pattern. RESET voltage of 2.5*V*, SET current of 55μ*A*, and programming time of 1μ*s*. **(b,c)** Proportion of 1 values read by the on-chip precharge sense amplifier, over 100 whole-array programming cycles of a memory array, for the two complementary checkerboards configuration. **(d)** Rate of programming failure indicated of the precharge sense amplifier circuits as a function of the ratio between HRS and LRS resistance (measured by a sense measure unit) in the same configuration as **(a–c)**. **(e–f)** Proportion of 1 values read by the on-chip precharge sense amplifier, over 100 whole-array programming cycles of a memory array, for the last layer of a binarized neural network trained on MNIST (details in body text).

This section first presents the results of electrical characterization of the differential OxRAM arrays. We program the array with checkerboard-type data, alternating zero and one bits, using programming times of one microsecond. For programming devices in HRS (RESET operation), the access transistor is fully opened, and a reset voltage of 2.5*V* is used. For programming devices in LRS (SET operation), the gate voltage of the access transistor is chosen to ensure a compliance current of 55μ*A*. Figure 4a shows the statistical distribution of the LRS and HRS of the cells, based on 100 programming cycles of the full array. This graph uses a standard representation in the memory field, where the *y* axis is expressed as the number of standard deviations of the distribution (Ly et al., 2018). The figure superimposes distributions of left (BL) and right (BLb) columns of the array, and no significant difference is seen between BL and BLb devices. The LRS and HRS distributions are clearly separate but overlap at a value of three standard deviations, which makes bit errors possible. If a 1T1R structure were used, a bit error rate of 0.012 (1.2%) would be seen with this distribution. By contrast, at the output of the precharge sense amplifiers, a bit error rate of 0.002 (0.2%) is seen, providing a first indication of the benefits of the 2T2R approach. Figures 4b,c show the mean error (using the 2T2R configuration) on the whole array for the two types of checkerboards. We see that all devices can be programmed in HRS and LRS. A few devices have an increased bit error rate. This graph highlights the existence of both cycle-to-cycle and device-to-device variability and the absence of “dead” cells.

We now validate in detail the functionality of the precharge sense amplifiers. The precise resistance of devices is first measured by deactivating the precharge sense amplifiers and using the external source monitor units. Then, the precharge sense amplifiers are reactivated, and a sense operation is performed. Figure 4d plots the mean measurement of the sense amplifiers as a function of the ratio between the two resistances that are being compared, superimposed on the ideal behavior of a sense amplifier. The sense amplifiers show excellent functionality but can make mistakes if the two resistances differ by less than a factor of five. Finally, Figures 4e,f show the results of repeating the experiments of Figures 4b,c in a more realistic situation and on a different die. We trained a memory array 100 times with weights corresponding to the last layer of a binarized neural network trained on the MNIST task of handwritten digit recognition. As in the checkerboard case, no dead cell is seen, and a similar degree of cycle-to-cycle and device-to-device variation is exhibited.

**Figure 5:**
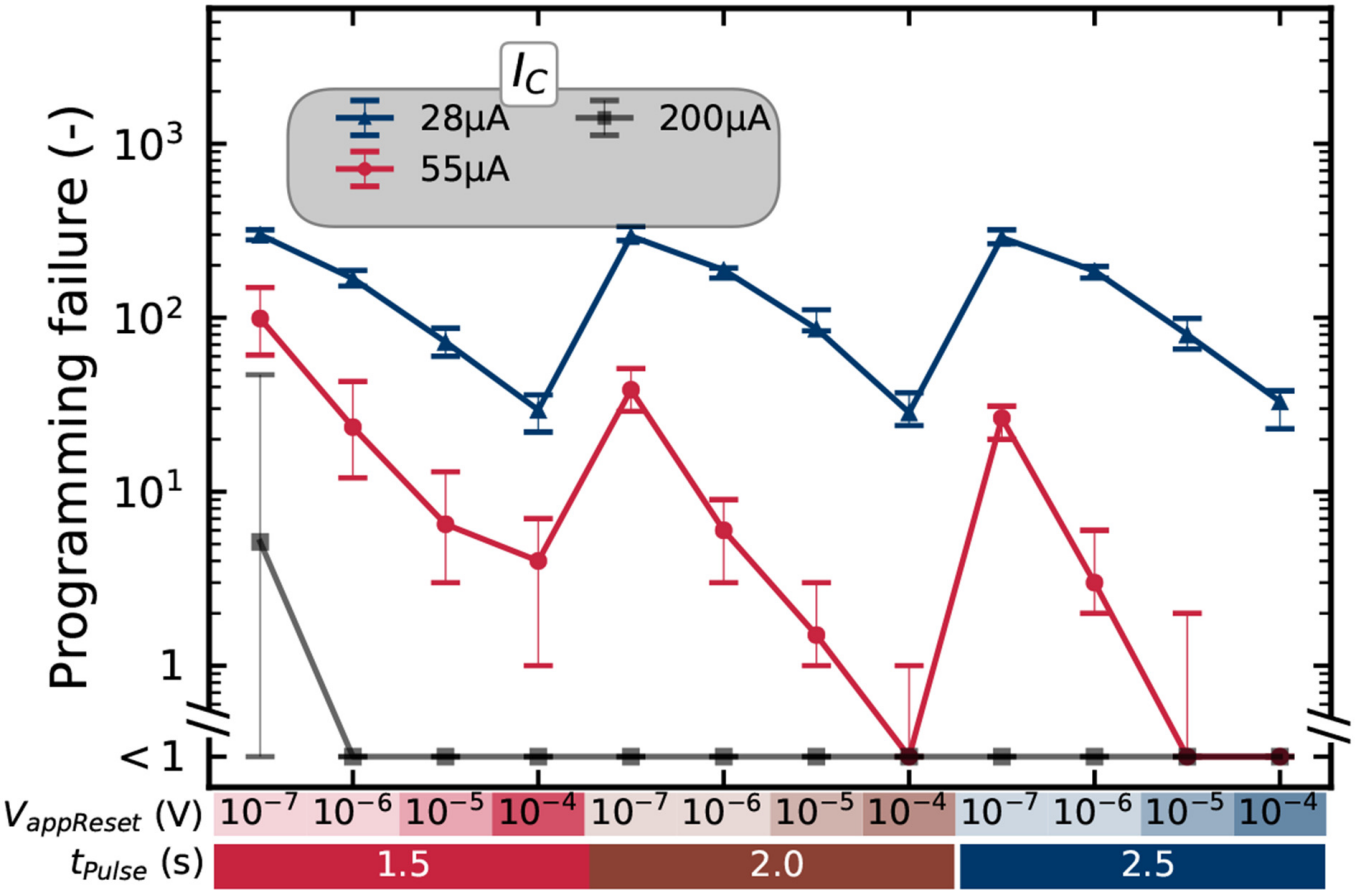
Number of errors for different programming conditions, as measured by precharge sense amplifier, for a 2T2R configuration on a kilobit memory array. The “ < 1” label means that no errors were detected. The error bars present the minimum and maximum number of detected errors over five repetitions of the experiments.

The programming rates are strongly dependent on the programming conditions. Figure 5 shows the mean number of incorrect bits on a whole array for various combinations of programming time (from 0.1 to 100μ*s*), RESET voltage (between 1.5 and 2.5 Volts), and SET compliance current (between 28 and 200μ*A*). We observe that the bit error rate depends extensively on these three programming parameters, the SET compliance current having the most significant impact.

**Figure 6:**
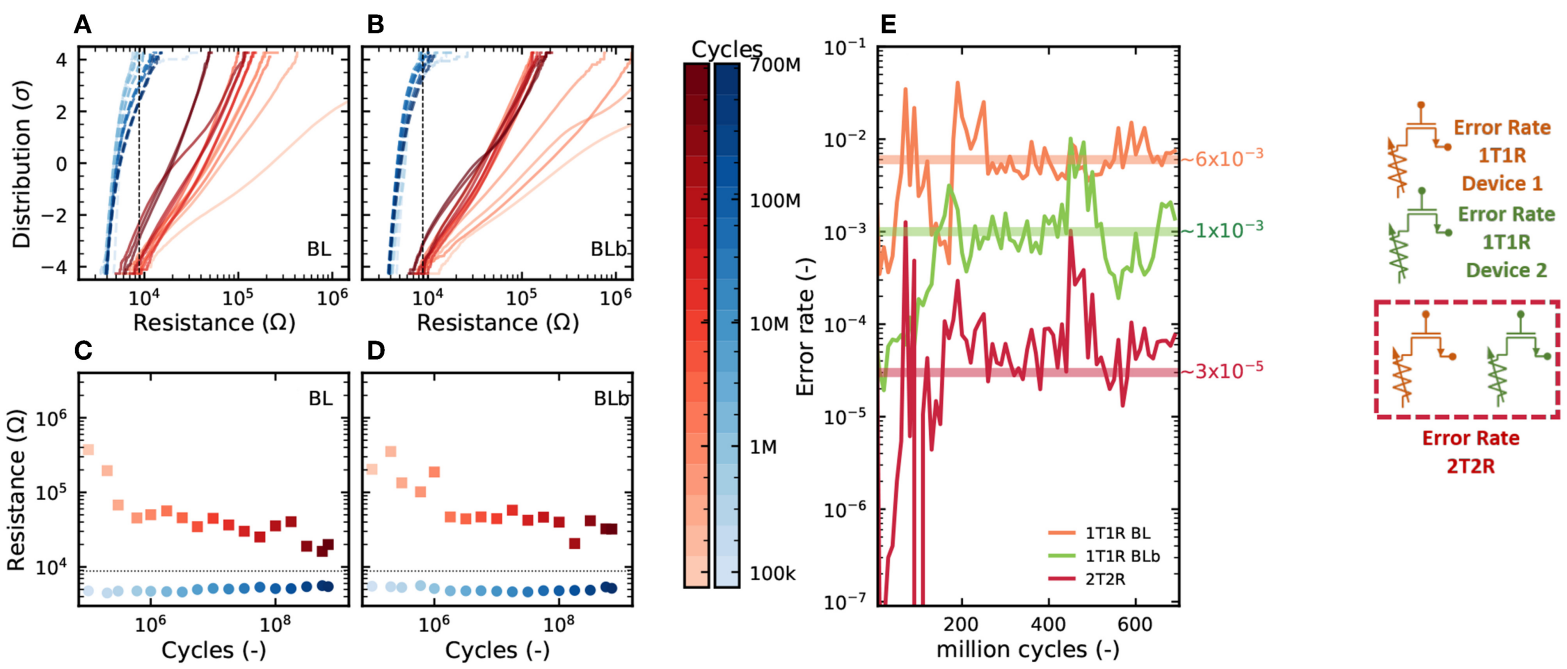
**(A,B)** Distribution of resistance values, **(C,D)** mean resistance value, and **(E)** mean bit error rate over 10 million cycles, as measured by precharge sense amplifier, in the 2T2R configuration, as a function of the number of cycles for which a device has been programmed. RESET voltage of 2.5*V*, SET current of 200μ*A*, and programming time of 1μ*s*.

**Figure 7:**
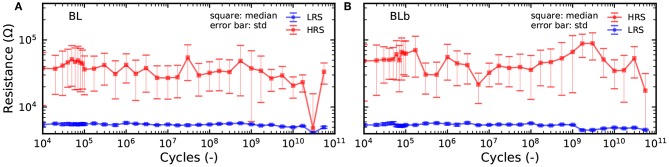
**(A,B)** Mean resistance value of the BL and BLb device over 10 thousand cycles for measurements of a device pair over 5 × 10^10^ cycles. RESET voltage of 1.5*V*, SET current of 200μ*A*, and programming time of 1μ*s*.

In Figure 6, we look more precisely at the effects of cycle-to-cycle device variability and device aging. A device and its complementary device were programmed through 700 million cycles. Figures 6A,B show the distribution of the LRS and HRS of the device under test and its complementary device after different number of cycles, ranging from the first one to the last one. We can observe that as the devices are cycled, the LRS and HRS distributions become less separated and start to overlap at a lower number of standard deviations. This translates directly to the mean resistance of the devices in HRS and LRS (Figures 6C,D), which become closer as the device ages. More importantly, the aging process impacts the device bit error rate (Figure 6E): the bit error rate of the device and its complementary device increase by several orders of magnitudes over the lifetime of the device. The same effect is seen on the bit error rate resulting from the precharge sense amplifier (2T2R), but it remains at a much lower level: while the 1T1R bit error rate goes above 10^−3^ after a few million cycles, the 2T2R remains below this value over the 700 million cycles. This result highlights that the concept of cyclability depends on the acceptable bit error rate and that the cyclability at constant bit error rate can be considerably extended by using the 2T2R structure. It should also be highlighted that the cyclability depends tremendously on the programming conditions. Figures 7A,B shows endurance measurements with a reset voltage of 1.5*V* (all other programming conditions are identical to Figures 6A–E). We can see that the device experiences no degradation through more than ten billion cycles. Over that time, the 2T2R bit error rate remains below 10^−4^.

**Figure 8:**
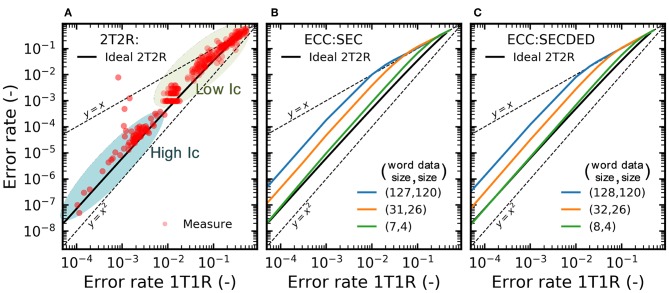
**(A)** Experimental bit error rate of the 2T2R array, as measured by precharge sense amplifier, as a function of the bit error rate obtained with individual (1T1R) RRAM devices under the same programming conditions. The detailed methodology for obtaining this graph is presented in the body text. Bit error rate obtained with **(B)** Single Error Correcting (SEC) and **(C)** Single Error Correcting Double Error Detection (SECDED) ECC as a function of the error rate of the individual devices.

We now aim at quantifying and benchmarking the benefits of the 2T2R structure more precisely. We performed extensive characterization of bit error rates on the memory array in various regimes. Figure 8A presents different experiments where the 2T2R bit error rate is plotted as a function of the bit error rate that would be obtained by using a single device programmed in the same conditions. The different points are obtained by varying the compliance current Ic during SET operations, and the graph associates two types of experiments:

- The points marked as “Low Ic” are obtained using whole-array measurement, where devices are programmed with a low SET compliance current to ensure a high error rate. Each device in the memory array is programmed once (following the checkerboard configuration), and all synaptic weights are read using the on-chip precharge sense amplifiers. The plotted bit error rate is the proportion of weights for which the read weight differs from the weight value targeted by the programming operation.
- The points marked as “High Ic” are obtained by measurements on a single device pair. A single 2T2R structure in the array is programmed ten million times by alternating programming to +1 and −1 values. The value programmed in the 2T2R structure is read using an on-chip precharge sense amplifier after each programming operation. The plotted bit error rate is the proportion of read operations for which the read weight differs from the targeted value.

We can see that the 2T2R bit error rate is always lower than the 1T1R one. The difference is larger for a lower bit error rate and reaches four orders of magnitude for a 2T2R bit error rate of 10^−8^. The black line represents calculations where the precharge sense amplifier is supposed to be ideal (i.e., to follow the idealized dotted characteristics of Figure 4C).

To interpret the results of the 2T2R approach in a broader perspective, we benchmark them with standard error-correcting codes. Figures 8B,C show the benefits of two codes, using the same plotting format as Figure 8A: a Single Error Correction (SEC) and a Single Error Correction Double Error Detection (SECDED) code, presented with different degrees of redundancy. These simple codes, formally known as Hamming and extended Hamming codes, are widely used in the memory field. Interestingly, we see that the benefit of these codes are very similar to the benefit of our 2T2R approach with an ideal sense amplifier, at equivalent memory redundancy (e.g., SECDED(8,4)), although our approach uses no decoding circuit and performs the equivalent of error correction directly within the sense amplifier. By contrast, ECCs can also reduce bit errors, to a lesser extent, using less redundancy, but the required decoding circuits utilize hundreds to thousands of logic gates (Gregori et al., 2003). In a context where logic and memory are tightly integrated, these decoding circuits would need to be repeated many times, and as their logic is much more complicated than that of binarized neural networks, they would be the dominant source of computation and energy consumption. ECC circuits are also incompatible with the idea of integrating XNOR operations within the sense amplifiers and cause important read latency.

### 3.2. Do All Errors Need to Be Corrected?

Based on the results of the electrical measurements, and before discussing the whole system, it is important to determine the OxRAM bit error rate levels that can be tolerated for applications. To answer this question, we performed simulations of binarized neural networks on four different tasks:

- MNIST handwritten digit classification (LeCun et al., 1998), the canonical task of machine learning. We use a fully connected neural network with two 1024-neuron hidden layers.
- The CIFAR-10 image recognition task (Krizhevsky and Hinton, 2009), which consists of recognizing 32 × 32 color images spread between ten categories of vehicles and animals. We use a deep convolutional network with six convolutional layers using kernels of 3 × 3 and a stride of one, followed by three fully connected layers.
- The ImageNet recognition task, which consists of recognizing 224 × 224 color images out of 1000 classes. This task is considerably more difficult than MNIST and CIFAR-10. We use the historic AlexNet deep convolutional neural network (Krizhevsky et al., 2012).
- A medical task involving the analysis of electrocardiography (ECG) signals: automatic detection of electrode misplacement. This binary classification challenge takes as input the ECG signals of twelve electrodes. The experimental trial data are sampled at 250 Hz and have a duration of three seconds each. To solve this task, we employ a convolutional neural network composed of five convolutional layers and two fully connected layers. The convolutional kernel (sliding window) sizes decrease from 13 to 5 in each subsequent layer. Each convolutional layer produces 64 filters detecting different features of the signal.

**Figure 9:**
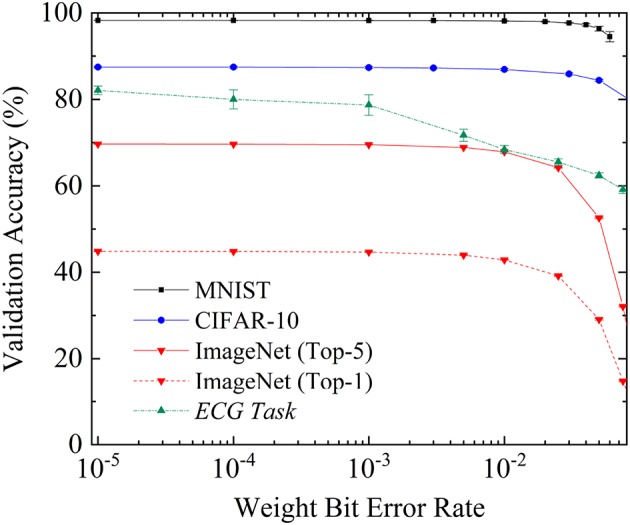
Recognition rate on the validation datasets of the fully connected neural network for MNIST, the convolutional neural network for CIFAR10, and AlexNet for ImageNet (Top 5 and Top 1) and in the ECG analysis task, as a function of the bit error rate over the weights during inference. Each experiment was repeated five times; the mean recognition rate is presented. Error bars represent one standard deviation.

Fully binarized neural networks were trained on these tasks on NVIDIA Tesla GPUs using Python and the PyTorch deep-learning framework. Once the neural networks were trained, we ran them on the dataset validation sets, artificially introducing errors into the neural network weights (meaning some +1 weights were replaced by −1 weights, and reciprocally). Using this technique, we could emulate the impact of OxRAM bit errors. Figure 9 shows the resulting validation accuracy as a function of the introduced bit error rate for the four tasks considered. In the case of ImageNet, both the Top-1 (proportion of validation images where the right label is the top choice of the neural network) and the Top-5 (proportion of validation images where the right label is within the top five choices of the neural network) are included.

On the three-vision tasks (MNIST, CIFAR-10, and ImageNet), we see that extremely high levels of bit errors can be tolerated: up to a bit error rate of 10^−4^, the network performs as well as with no errors. Minimal performance reduction starts to be seen with bit error rates of 10^−3^ (the Top-5 accuracy on ImageNet is degraded from 69.7% to 69.5%). At bit error rates of 0.01, the performance reduction becomes significant. The reduction is more substantial for harder tasks: MNIST accuracy is only degraded from 98.3% to 98.1%, CIFAR-10 accuracy is degraded from 87.5% to 86.9%, while ImageNet Top-5 accuracy is degraded from 69.7% to 67.9%.

The ECG task also shows extremely high bit error tolerance, but bit errors have an effect more rapidly than in the vision tasks. At a bit error rate of 10^−3^, the validation accuracy is reduced from 82.1% to 78.7%, and at a bit error rate of 0.01, to 68.4%. This difference between vision and ECG tasks probably originates in the fact that ECG signals carry a lot less redundant information than images. Nevertheless, we see that even for ECG tasks, high bit error rates can be accepted with regards to the standards of conventional digital electronics.

## 4. Discussion

### 4.1. Projection at the System Level

#### 4.1.1. Impact of In-Memory Computation

We now use all the paper results to discuss the potential of our approach. Based on our ASIC design, using the energy-evaluation technique described at the end of the Methods section, we find that our system would consume 25*nJ* to recognize one handwritten digit, using a fully connected neural network with two hidden layers of 1,024 neurons. This is considerably less than processor-based options. For example, Lane et al. (2016) analyses the energy consumption of inference on CPUs and GPUs: operating a fully connected neural network with two hidden layers of 1,000 neurons requires 7 to 100 millijoules on a low-power CPU (from NVIDIA Tegra K1 or Qualcomm Snapdragon 800 systems on the chip) and 1.3 millijoules on a low-power GPU (NVIDIA Tegra K1).

These results are not surprising due to the considerable overhead for accessing memory in modern computers. For example, Pedram et al. (2017) shows that accessing data in a static RAM cache consumes around fifty times more energy than the integer addition of this data. If the data is stored in the external dynamic RAM, the ratio is increased to more than 3,000. Binarized Neural Networks require minimal arithmetic: no multiplication and only integer addition with a low bit width. When operating a Binarized Neural Network on a CPU or GPU, almost the entirety of the energy is used to move data, and the inherent topology of the neural network is not exploited to reduce data movement. Switching to in-memory or near-memory computing approaches therefore has the potential to reduce energy consumption drastically for such tasks. This is especially true as, in inference hardware, synaptic weights are static and can be programmed to memory only once if the circuit does not need to change function.

#### 4.1.2. Impact of Binarization

We now look specifically at the benefits of relying on Binarized Neural Networks rather than real-valued digital ones. Binarized Neural Networks feature considerably simpler architecture than conventional neural networks but also require an increased number of neurons and synapses to achieve equivalent accuracy. It is therefore essential to compare the binarized and real-value approaches.

Most digital ASIC implementations of neural networks have an inference function with eight-bit fixed-point arithmetic, the most famous example being the tensor processing units developed by Google (Jouppi et al., 2017). At this precision, no degradation is usually seen for inference with regards to 32- and 64-bis floating-point arithmetic.

**Figure 10:**
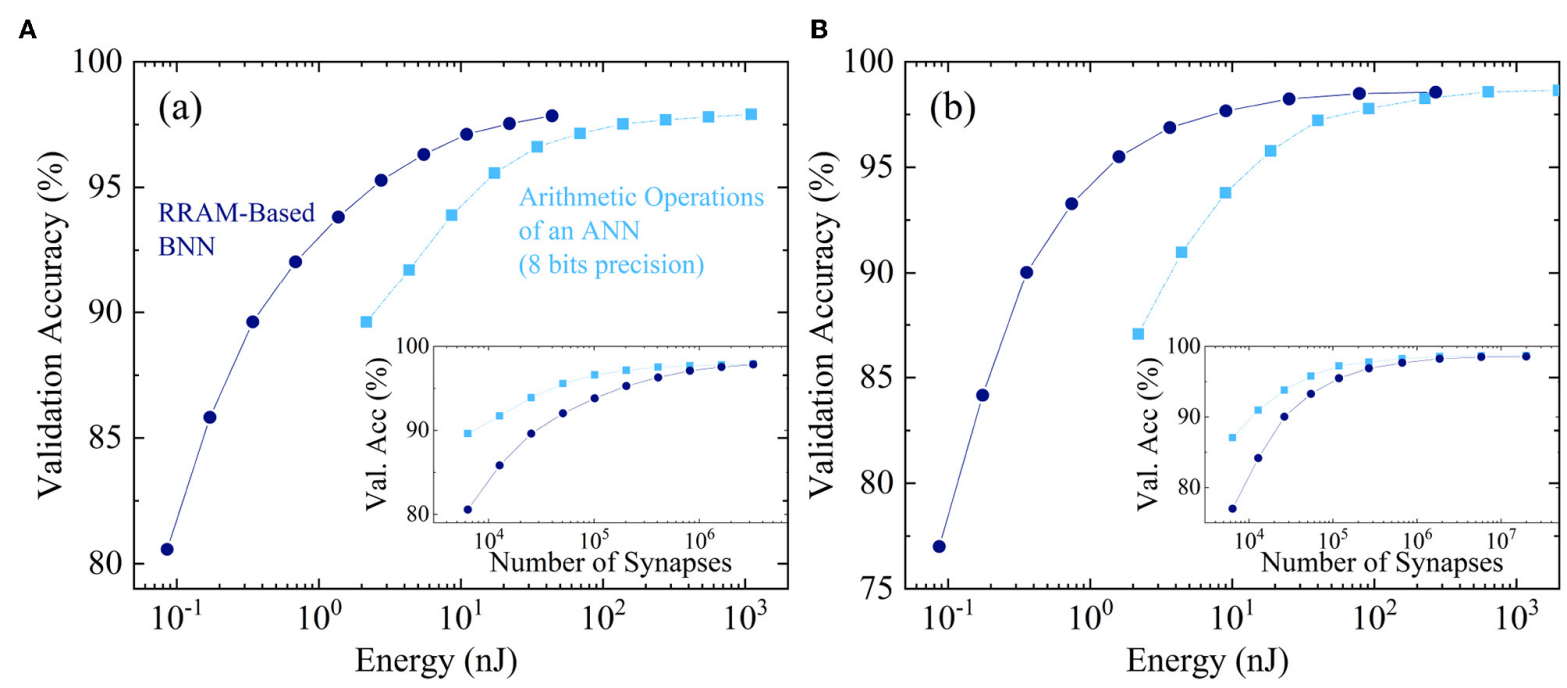
Dark blue circles: MNIST validation accuracy as a function of the inference energy of our Binarized Neural Network hardware design. Light blue squares: same, as a function of the energy used for arithmetic operation in a real-valued neural network employing eight-bit fixed-point arithmetic. The different points are obtained by varying the number of hidden neurons in **(A)** a one hidden layer neural network and **(B)** a two hidden layers neural network. Insets: number of synapses in each situation.

To investigate the benefits of Binarized Neural Networks, Figure 10 looks at the energy consumption for inference over a single MNIST digit. We consider two architectures: a neural network with a single hidden layer (Figure 10A) and another with two hidden layers (Figure 10B), and we vary the number of hidden neurons. Figures 10A,B plot on the *x*-axis the estimated energy consumption of a Binarized Neural Network using our architecture based on the flow presented in the Methods section. It also plots the energy required for the arithmetic operations (sum and product) of an eight-bit fixed-point regular neural network, neglecting the overhead that is considered for the Binarized Neural Network. For both types of networks, the *y*-axis shows the resulting accuracy in the MNIST task. We see that at equivalent precision, the Binarized Neural Network always consumes less energy than the arithmetic operations of the real-valued one. It is remarkable that the energy benefit depends significantly on the targeted accuracy and should therefore be investigated on a case-by-case basis. The highest energy benefits, a little less than a factor ten, are seen at lower targeted precision.

Binarized Neural Networks have other benefits with regards to real-valued digital networks: if the weights are stored in RRAM, the programming energy is reduced due to the lower memory requirements of Binarized Neural Networks. The area of the overall circuit is also expected to be reduced due to the absence of multipliers, which are high-area circuits.

#### 4.1.3. Comparison With Analog Approaches

As mentioned in the introduction, a widely studied approach for implementing neural networks with RRAM is to rely on an analog electronics strategy, where Ohm's law is exploited for implementing multiplications and Kirchoff's current law for implementing additions (Prezioso et al., 2015; Serb et al., 2016; Shafiee et al., 2016; Ambrogio et al., 2018; Li et al., 2018; Wang et al., 2018). The digital approach presented in this paper cannot be straightforwardly compared to the analog approach: the detailed performance of the analog approach depends tremendously on its implementation details, device specifics, and the size of the neural network. Nevertheless, several points can be raised.

First, the programming of the devices is much simpler in our approach than in the analog one: one only needs to program a device and its complementary device in LRS and HRS, which can be achieved by two programming pulses. It is not necessary to verify the programming operation, as the neural network has inherent bit-error tolerance. Programming RRAM for analog operation is a more challenging task and usually requires a sequence of multiple pulses (Prezioso et al., 2015), which leads to higher programming energy and device aging.

For the neural network operation, the analog approach and ours function differently. Our approach reads synaptic values using the sense amplifier, which is a highly energy-efficient and fast circuit that can operate at hundreds of picoseconds in advanced CMOS nodes (Zhao et al., 2014). This sense amplifier inherently produces the multiplication operation, and then the addition needs to be performed using a low bit-width digital integer addition circuit. The ensemble of a read operation and the corresponding addition typically consumes fourteen femtojoules in our estimates in advanced node. In the analog approach, the read operation is performed by applying a voltage pulse and inherently produces the multiplication through Ohm's law but also the addition though Kirchoff law. This approach is attractive, but, on the other hand, requires the use of CMOS analog overhead circuitry such as an operational amplifier, which can bring high energy and area overhead. Which approach is the most energy-efficient between ours and the analog one will probably depend tremendously on memory size, application, and targeted accuracy.

Another advantage of the digital approach is that it is much simpler to design, test, and verify as it relies on all standard VLSI design tools. On the other hand, an advantage of the analog approach is that it may, for a small memory size, function without access transistors, resulting in higher memory densities (Prezioso et al., 2015).

#### 4.1.4. Impact in Terms of Programming Energy and Device Aging

A last comment is that the bit error tolerance of binarized neural networks can have considerable benefits at the system level. Table 1 summarizes the measured properties of RRAM cells under different programming conditions, chosen from those presented in Figure 5. We consider only the conditions with bit error rates below 10^−3^ (i.e., corresponding to a “ <1” data point in Figure 5), as this constraint makes them appropriate for use for all tasks considered in section 3.2. The “strong” programming conditions are the ones presented in Figure 6. They feature a low bit error rate before aging but high programming energy. The other two columns correspond to two optimized choices. The conditions optimized for programming energy are the conditions of Figure 5 with bit error rates below 10^−3^ and the lowest programming energy. They use a lower RESET voltage (2.0*V*) than the strong conditions and a shorter programming time (100*ns*). The cyclability of the device—defined as the number of programming cycles a cell can perform while retaining a bit error rate below 10^−3^—remains comparable to the strong programming conditions. The conditions optimized for endurance are, by contrast, the conditions of Figure 5 with a bit error rate below 10^−3^ and the highest cyclability: more than 10^10^ cycles, as already evidenced in Figure 7. These conditions use a low RESET voltage 1.5*V* but require a programming time of 1μ*s*.

**Table 1:** RRAM Properties with different programming conditions.

**Programming condition**	**Strong (Figure 6)**	**Optimized endurance (Figure 7)**	**Optimized programming energy**
SET compliance current	200μ*A*	200μ*A*	200μ*A*
RESET voltage	2.5*V*	1.5*V*	2*V*
Programming time	1μ*s*	1μ*s*	100*ns*
2T2R bit error rate (before aging)	< 10^−7^	< 10^−4^	< 10^−5^
Programming energy	200 ~ 400*pJ*	150 ~ 400*pJ*	20 ~ 30*pJ*
(SET/RESET)			
Cyclability (number of cycles at BER < 10^−3^)	> 10^8^	> 10^10^	> 10^8^

### 4.2. Conclusion

This work proposes an architecture for implementing binarized neural networks with RRAMs and incorporates several biologically plausible ideas:

- Fully co-locating logic memory,
- Relying only on-low precision computation (through the Binarized Neural Network concept),
- Avoiding multiplication altogether, and
- Accepting some errors without formal error correction.

At the same time, our approach relies on conventional microelectronics ideas that are non-biological in nature:

- Relying on fixed-point arithmetic to compute sums, whereas brains use analog computation,
- Using sense amplifier circuits, which are not brain-inspired, and
- Using a differential structure to reduce errors, a traditional electrical engineering strategy.

Based on these ideas, we designed, fabricated, and extensively tested a memory structure with its peripheral circuitry and designed and simulated a full digital system based on this memory structure. Our results show that this structure allows neural networks to be implemented without the use of Error-Correcting Codes, which are usually used with emerging memories. Our approach also features very attractive properties in terms of energy consumption and can allow that use of RRAM devices in a “weak” programming regime, where they have low programming energy and outstanding endurance. These results highlight that although in-memory computing cannot efficiently rely on Error-Correcting Codes, it can still function without stringent requirements on device variability if a differential memory architecture is chosen.

When working on bioinspiration, drawing the line between bio-plausibility and embracing the differences between the nanodevices of the brain and electronic devices is always challenging. In this project, we highlight that digital electronics can be enriched by biologically plausible ideas. When working with nanodevices, it can be beneficial to incorporate device physics questions into the design, and not necessarily to target the level of determinism that we have been accustomed to by CMOS.

This work opens multiple prospects. On the device front, it could be possible to develop more integrated 2T2R structures to increase the density of the memories. The concept of this work could also be adapted to other kinds of emerging memories, such as phase-change memories and spin torque magnetoresistive memories. At the system level, we are now in a position to fabricate larger systems and to investigate the extension of our concept to more varied forms of neural network architecture such as convolutional and recurrent networks. In the case of convolutional networks, a dilemma appears between taking the in-memory computing approach to the fullest degree, by replicating physically convolutional kernels or implementing some sequential computation to minimize resources, as works have started to evaluate already. These considerations open the way for truly low-energy artificial intelligence for both servers and embedded systems.

## Data Availability Statement

The datasets generated for this study are available on request from the corresponding author.

## Author Contributions

EV and EN were in charge of fabrication and of the initial RRAM characterization. J-MP performed the CMOS design of the memory array. MB performed the electrical characterization. TH designed the BNN systems. TH, BP, and DQ performed the BNN simulations. DQ wrote the initial version of the paper. J-OK, EV, J-MP, and DQ planned and supervised the project. All authors participated in data analysis and the writing of the paper.

### Conflict of Interest

The authors declare that the research was conducted in the absence of any commercial or financial relationships that could be construed as a potential conflict of interest.
